# Assessment on Knowledge Network Sharing Capability of Industrial Cluster Based on Dempster-Shafer Theory of Evidence

**DOI:** 10.1155/2014/810782

**Published:** 2014-03-24

**Authors:** Shengli Dai, Hailin Zhang

**Affiliations:** ^1^School of Public Management, Central China Normal University, 152 Luoyu Road, Wuhan 430079, China; ^2^Local Governance and Local Development Research Centre of Hubei, Central China Normal University, 152 Luoyu Road, Wuhan 430079, China; ^3^School of Urban and Environmental Sciences, Central China Normal University, 152 Luoyu Road, Wuhan 430079, China

## Abstract

Based on Theory of Evidence and reviewing research papers concerned, a concept model of knowledge sharing network among industrial cluster firms, which can be applied to assess knowledge sharing capacity, has been built. Next, the authors create a set of assessment index systems including twelve subindexes under four principle indexes. In this study, ten experts in the same field were invited to score all the indexes of knowledge sharing capacity concerning one certain industrial cluster. The research result shows relatively high knowledge network sharing capacity among the certain industrial cluster firms. Another conclusion is that the assessment method with Theory of Evidence is feasible to conduct such a research.

## 1. Introduction

In recent years, it has become increasingly apparent that cluster economy, accompanied by industrial cluster boom, is one important form of the world economy development and presents a state that develops quickly. In the study field of industrial cluster, operation of knowledge network is focused on by some researchers.

The relationship of cooperation between enterprises and knowledge exchange attracts more and more attention. In the study of process of knowledge transfer, emphasis is widely put on knowledge characteristic (e.g., recessive, tacit-codified, individual-social, appropriable-exclusive, and divisible-indivisible) [1]. There is also much research, which contributes to knowledge network of industrial cluster. The Framework of Knowledge Networks comprises the following components: actors individuals, groups, and organizations; relationships between actors, which can be categorized by form, content, and intensity; resources which may be used by actors within their relationships, and institutional properties, including structural and cultural dimensions such as control mechanisms, standard operating procedures, norms and rules, and communication patterns [2]. Regional clusters depend on the networks that arise from reciprocal linkages among colocated organizations, while physical proximity among firms can alter the nature of information and resource flows through networks [3]. In all industries, knowledge can flow through the linkage border and the effect of knowledge flow is enhanced by spatial proximity between the actors taking part in the exchange [4]. The focal firms and their organized networks within industrial districts, being the driving force of cluster innovation, play a crucial role in the process of knowledge creation and transfer [5]. Knowledge alliance, one kind of strategic alliance similar to knowledge network, means a group of firms entering into voluntary arrangements that involve knowledge creation, transfer and exchange [6]. Alliances may serve different purposes from knowledge alliance formation. However, rather than using alliances to acquire capabilities, scholars suggest that firms use interfirm collaboration to gain access to other firms' capabilities, supporting more focused, intensive exploitation of existing capabilities [7]. Based on heterogeneity of knowledge acquired from accumulative research, different firms can develop various capabilities [8]. Some researches concerning knowledge transfer and sharing have been done. The study of Ahuja and Giuliani shows the existing relation between structural properties of social networks and learning and innovation output [9, 10]. Ahuja's study shows that direct and indirect ties between a firm's partners both have a positive impact on innovation but that the impact of indirect ties is moderated by the number of a firm's direct ties. More brokerage opportunities in network firms relate to better innovative performance, because actors in a network rich in structural holes will be able to access novel information from remote parts of the network and exploit that information to their advantage. Hansen's research argues that indirect ties in network firms are conducive to knowledge transfer and focal firms usually are the bridge of the connections [11]. In the study of the Boston Biotechnology Community innovative activities, Owen-Smith and Powell emphasize that focal firms are the organizer of the network knowledge and fundamentally alter the flow of information through a network [12, 13].

Though there are many insights on explaining the process of knowledge transfer and innovation, more emphasis was put on structural character of knowledge network. Because of much uncertainty, many methods have been applied to evaluate the capacity of knowledge networks sharing, for example, Analytic Hierarchy Process, Fuzzy Theory, Grey Theory, Neutral Network Analysis, Data Envelop Analyze, Factor Analysis, and Bayesian Network. In our view, the existing researches on assessing the capacity of knowledge networks sharing have a number of important weaknesses and constraints, especially on assessment methodologies and unclear attribute of the evaluation objects. In this research, to begin with, one new evaluation index system concerning the evaluation object will be established. We attempt to assess the capacity of knowledge networks sharing among cluster industries based on another powerful method—Dempster-Shafer Theory of Evidence.

**Figure 1:**
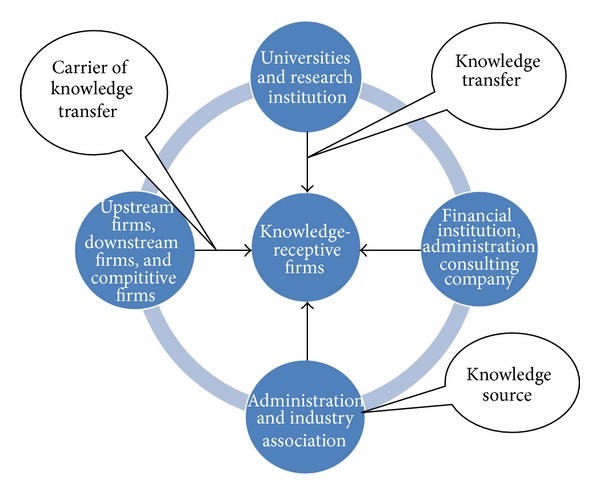
Flowchart of knowledge network sharing among cluster industries.

There are quite a few models concerning knowledge networks sharing among cluster industries. Szulanski argues that knowledge transfer process includes four stages—initiation, implementation, ramp-up, and integration [14]. In Xiao's opinion, there are several categories of Knowledge Flows in Knowledge Networks: technical corporation, talent flow, new technology transfer, transfer of patent right, market research, and informal exchange [15]. Zhang constructs a cluster model of enterprise knowledge networks based on social network tools and argues that knowledge base, recessive knowledge center, knowledge transfer center, knowledge innovation center, and knowledge expansion center exist among cluster enterprises [16]. Above, researches present a reasonable analysis on operation mechanism of knowledge network sharing among cluster industries and draw some valuable conclusions. In authors' view, much efficient discussion on the knowledge sharing process is required. In the knowledge sharing process among cluster industries, knowledge from knowledge source, combined with knowledge transfer carrier, finally transfers to knowledge accepter via certain transfer path. The knowledge source includes universities, institutions, financial institutions, administration consulting company, administration and industry association, upstream firms, downstream firms, and competitive firms as shown in Figure 1.

## 2. Computational Methods for Evidence Theory

Dempster-Shafer theory of evidence was put forward on early 1976 by Dempster and developed by Shafer [17]. Generally, evidences are conclusions of one specific research and observation, which is one part of experience and knowledge. Based on evidences, initial allocations are created, which means to calculate the degrees of support on each proposition. In actual study, evidences available can be used to calculate collective contribution to every proposition and this method can effectively solve the problem of uncertainty in knowledge network sharing assessment.

Generally, suppose that one sampling space is called a frame of discernment  *θ*, one proposition  *H*_*q*_(*q* = 1,2,…, *z*) is subset of set  *θ*, and *H*_*q*_ is individually independent. In the study, some definitions of belief allocation, belief function, and focal elements are brought forward. Evidence combination rules are introduced and knowledge network sharing capability of cluster industries is assessed.

Given a question of interest, let  *θ* be a finite set of possible answers to the question, called a frame of discernment, and let 2^*θ*^ be the set of all subsets of  *θ*:

(1) $$\begin{array}{c} 2^{\theta }=\left\{A\;|\;A\subseteq \theta \right\}. \end{array}$$

The subset *A* includes as special cases the empty set *φ* and the full set  *θ*. It represents a statement or proposition that the truth lies in *A*. A real function over the subsets Bel:  2^*θ*^ → [0, 1] is called a belief function if and only if it satisfies the following three axioms:

(2) (a)  m(f)=0(b)  ∑A⊂θm(A)=1, ∀A⊆θ,

where *m* is defined as basic probability on frame of discernment  *θ and m*(*A*)* is called Proposition A's basic probability number.* The vacuous belief function is a simple support function with focus *θ*. If Bel is a simple support function with focus *F* = *θ*, then *m*(*F*) = *s*, *m*(*θ*) = 1 − *s*, and *m* is 0 elsewhere. Thus, a simple support function invests all of our committed belief on the disjunction represented by its focus, *F*, and all our uncommitted belief on *θ*. Consider

(3) $$\begin{array}{c} \text{Bel}\left(A\right)=\underset{B\subset A}{\sum }m\left(B\right)\;\left(\forall A\subset \theta \right). \end{array}$$

The subsets *A* of  *θ* such that *m*(*A*) > 0 are called the focal elements of the belief function, and their union is called its core.

Based on the definitions above, evidence combination rules can be derived. Let *m*_1_ and *m*_2_ be basic probability assignments on the same frame  *θ*. The function is defined as

(4) $$\begin{array}{c} m_{12}\left(f\right)=0,\\ m_{12}\left(A\right)=\frac{1}{1-k}\underset{X\cap Y=A}{\sum }m_{1}\left(X\right)m_{2}\left(Y\right),\;A\neq f, \end{array}$$

where *m*_12_ is a set function over the frame of discernment  *θ* and *K* = (1/(1 − *k*))∑_*X*∩*Y*=*f*_ *m*_1_(*X*)*m*_2_(*Y*) is conflict factor.

This formulation (4), called Dempster's rule of combination, namely, *m*_12_ = *m*_1_ · *m*_2_, represents the degrees of support by two independent evidences.

In the assessment process, firstly, hierarchy factors' values of model are scored by experts and decision makers and basic probabilities are obtained. Secondly, the assessment is completed based on the combination rules of evidence. There are five steps to assess knowledge network sharing capability among cluster industries via Evidence Theory. Consider the following.

(a) Based on analytic hierarchy process (AHP), the numerical weight is derived for each element of any hierarchy. Corresponding basic probability (*β*_*H*_*q*_(*e*_*jk*_^*i*^)_) of the lowest hierarchy elements (*e*_*jk*_^*i*^) over set *H*_*q*_ (*q* = 1,2,…, *z*) is acquired by experts and decision makers.
(b) Basic probability of each element of each hierarchy is computed. The elements with greatest weight is called core element and their probability over set *H*_*q*_ is *m*(*H*_*q*_ | *e*_*jk*_^*i*^) = *α*_*jk*_ *β*_*H*_*q*__(*e*_*jk*_^*i*^), while probability of the lowest elements over set *H*_*q*_ is *β*_*H*_*q*_(*e*_*jk*_^*i*^)_, where *α*_*jk*_ is preference coefficient reflecting experts' attitude to core elements. *α*_*jk*_'s value ranges over 0.9 ≤ *α*_*jk*_ ≤ 1. The greater is the *α*_*jk*_'s value, the more important is the core element. As to noncore elements, their basic probability over set *H*_*q*_ is expressed as *m*(*H*_*q*_ | *e*_*jk*_^*i*^) = (*w*_*jk*_/*w*_*jm*_)*α*_*jk*_ *β*_*H*_*q*__(*e*_*jk*_^*i*^), where *w*_*jm*_ is the weight of core element and *α*_*jk*_(*w*_*jk*_/*w*_*jm*_) is the element *e*_*jk*_^*i*^'s normalized weight. And the basic probability of the element *e*_*jk*_^*i*^ under completely uncertain is expressed as *m*(*H*_*q*_ | *e*_*jk*_^*i*^) = 1 − ∑_*q*=1_^*z*^ *m*(*H*_*q*_ | *e*_*jk*_^*i*^).
(c) Suppose that there is an element set *I*_(*n*)_ = {*e*_*i*1_, *e*_*i*2_,…,*e*_*in*_} within subsystem *I*_(*n*)_ = {*e*_*i*1_, *e*_*i*2_,…,*e*_*in*_}. Based on the combination rules, complex set function is generated as *m*_*I*_(*n*)__^*q*^ = *m*(*H*_*q*_ | *I*_(*n*)_) and *m*_*I*_(*n*)__^*θ*^ = *m*(*H*_*q*_ | *I*_(*n*)_), where *m*_*I*_(*n*)__^*q*^ represents the basic probability of all elements in the set *I*_(*n*)_ over set *H*_*q*_ and *m*_*I*_(*n*)__^*θ*^ represents the basic probability of the elements in the set *I*_(*n*)_ under completely uncertainty. When *n* = 2, *I*_(2)_ = {*e*_*i*1_, *e*_*i*2_}, equations will be induced by (4) as
  (5) $$\begin{array}{c} m_{I_{\left(2\right)}}^{q}=k_{I_{\left(2\right)}}\left[m_{1}^{q}m_{2}^{q}+m_{1}^{q}m_{2}^{\theta }+m_{1}^{\theta }m_{2}^{q}\right],\;q=1,2,\ldots ,z\\ m_{I_{\left(2\right)}}^{\theta }=k_{I_{\left(2\right)}}m_{1}^{\theta }m_{2}^{\theta }\\ k_{I_{\left(2\right)}}={\left(1-\overset{z}{\underset{t=1}{\sum }}\underset{q\neq t}{\sum }m_{1}^{t}m_{2}^{q}\right)}^{-1}. \end{array}$$
  Since *m*_*I*_(1)__^*q*^ = *m*_1_^*q*^ (*q* = 1,2,…, *z*) and *m*_*I*_(1)__^*θ*^ = *m*_1_^*θ*^, general term formula of recurrent sequence of *I*_(*p*+1)_ = {*e*_*i*1_, *e*_*i*2_,…,*e*_*ip*_, *e*_*i*(*p*+1)_} (*p* = 1,2,…, *z* − 1) is obtained through comprehensive induction as formulation (5) as

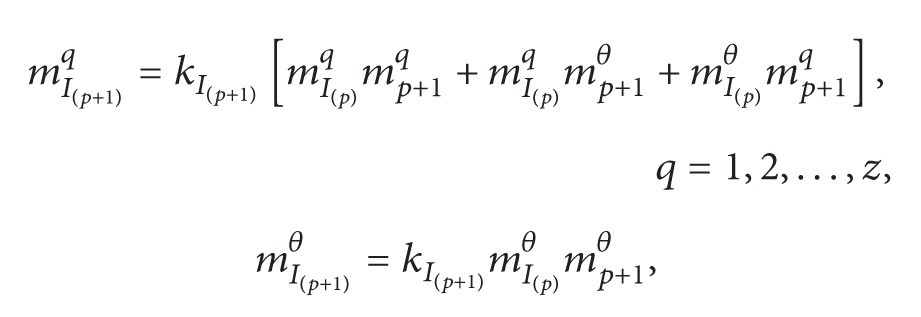
(6)

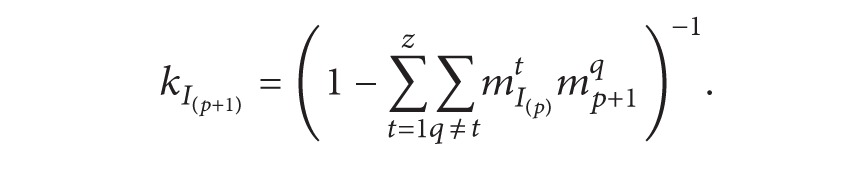
(7)
In the formulation (7), when *p* = *n* − 1, the set function of all the lowest hierarchy elements in subsystem *e*_*i*_ is obtained
  (8) $$\begin{array}{c} m\left(\left\{H_{q}\right\}\right)=m_{I_{\left(n\right)}}^{q},\;q=1,2,\ldots ,z,\\ m\left(H\right)=m_{I_{\left(n\right)}}^{\theta } \end{array}$$
  And *m*(*A*) = 0,   for all  *A* ≠ {*H*_*q*_}, *q* = 1,2,…, *z*,  *A* ≠ *H*.
(d) In the same way, the whole assessment system's set function can be obtained from formulations (6), (7), and (8); namely, the belief assignment of the whole system over set assessment set *H* = {*H*_1_, *H*_2_,…, *H*_*z*_}  (*q* = 1,2,…, *z*) is *m* ^*q*^(*q* = 1,2,…, *z*).
(e) Finally, formulation *S* = ∑_*q*=1_^*z*^ *m* ^*q*^ *p*(*H*_*q*_) is used to compute knowledge network sharing capability among cluster industries. The output is the result of evidential reasoning [18].

## 3. Construction of Assessment Indicator

In authors' view, there are several direct factors that affect the capability of knowledge network sharing among cluster industries. According to the model in the flowchart above, knowledge source, the pathway of knowledge transfer, carrier of knowledge transfer, and knowledge recipient are four prominent factors upon the capability of knowledge network sharing among cluster industries. Generally, the knowledge sharing capability of knowledge source is affected by vigor of knowledge, knowledge quantity, interest motive of knowledge sharing, and knowledge compatibility. The pathway of knowledge transfer is affected by remunerative transfer of new technology, talent flow, and technical corporation. Carrier of knowledge transfer is affected by competency of staff and knowledge sharing technology. Knowledge recipient is affected by knowledge deficiency, knowledge absorptive capacity, and willingness of receiving knowledge. Based on the factors above, the assessment index system is built. *V*_*i*_ represents the indicator and *w*_*i*_ represents the corresponding indicator's weight. Ten experts are invited to score each indicator and scores are listed in Table 1.

## 4. Weight of Assessment Indicator

In the aspect of weight, fuzzy clustering analysis is applied to avoid experts' subjective factors. Essentially, fuzzy clustering analysis is one classification method, which divides data elements into classes or clusters so that items in the same class are as similar as possible, and items in different classes are as dissimilar as possible. Under different supposed thresholds *λ*, different weights can be calculated as below.

### 4.1. Data Normalization

Given a universe *U* = {*u*_1_, *u*_2_,…,*u*_*m*_}, any index *u*_*i*_ is expressed by membership degree of *n* samples; namely, *u*_*i*_ = {*X*_*i*1_ = *μ*_1_(*u*_*i*_), *X*_*i*2_ = *μ*_2_(*u*_*i*_),…*X*_*ij*_ = *μ*_*j*_(*u*_*i*_),…*X*_*in*_ = *μ*_*n*_(*u*_*i*_)}. Then, the initial assessment matrix can be obtained,

(9) $$\begin{array}{c} X=\left\{X_{ij}=\mu_{j}\left(u_{i}\right)\right\}\;\left(i=1,2,\ldots m;j=1,2,\ldots n\right). \end{array}$$

And the matrix of normalized data (*Y*) is derived, *Y* = {*Y*_*ij*_}_*m*×*n*_, where *Y*_*ij*_ = *X*_*ij*_/∑_*j*=1_^*n*^ *X*_*ij*_ is normalization data of *x*_*ij*_ and *x*_*ij*_ is the simple value of the variable *i* measured in the level *j*.

### 4.2. Computation of Fuzzy Similar Matrix

Angle cosine method is applied to obtain fuzzy similar matrix *R* = (*r*_*ij*_), where *r*_*ij*_ represents relation coefficient between any two observed variables,

(10) $$\begin{array}{c} r_{ij}=\frac{\sum_{k=1}^{m}{X_{ik}X}_{jk}}{\sqrt{\sum_{k=1}^{m}{\left(X_{ik}\right)}^{2}{\left(X_{jk}\right)}^{2}}}, \end{array}$$

where *X*_*ik*_ and  *X*_*jk*_, respectively, represent the average scores assessed by ten experts, which range over 0 ≤ *X*_*jk*_ ≤ 1.

### 4.3. Construction of Fuzzy Equivalence Matrix

Convolution computation on fuzzy similar matrix is conducted after iterations until *R* ^*n*^ *R* = *R* ^*n*^ and *R* ^*k*^ = *R*2^*k*^ appear, and then fuzzy classification matrix is obtained.

### 4.4. Clustering

Given different threshold *λ* values, Boolean matrixes, which relate to the significance of the indictor, are obtained by computation of fuzzy classification matrix. Generally, the classification categories rise with the increase of *λ* value. In order to distinguish the significance of the assessment indicators, greater *λ* value should be chosen so that sequence and classification can be well done. Based on the different grades of *λ* values, weights of the assessment indicators are determined after data normalization.

## 5. Empirical Study

As described above, ten experts anticipate to score each assessment indicator and scores are listed in Table 1. Fuzzy clustering analysis is applied to determine the weight of each assessment indicator as *W* = (*W*_1_, *W*_2_, *W*_3_, *W*_4_, *W*_5_) = (0.4,0.2,0.1,0.3,0),  *W*_1_ = (*W*_11_, *W*_12_, *W*_13_, *W*_14_) = (0.35,0.25,0.2,0.2), *W*_2_ = (*W*_21_, *W*_22_, *W*_23_) = (0.4,0.3,0.3), *W*_3_ = (*W*_31_, *W*_32_) = (0.6,0.4), and *W*_4_ = (*W*_41_, *W*_42_, *W*_43_)  =  (0.3,0.5,0.2).* Let *preference coefficient be *α* = 0.9; then mass function about the indicator *V*_11_ over the fuzzy set *H* is computed. Vigor of knowledge is the core indicator of knowledge source and outputs are *m*(*H*_1_/*V*_11_) = 0.9 × 0.2 = 0.18, *m*(*H*_2_/*V*_11_) = 0.9 × 0.4 = 0.36, *m*(*H*_3_/*V*_11_) = 0.9 × 0.1 = 0.09, *m*(*H*_4_/*V*_11_) = 0.9 × 0.3 = 0.27, and *m*(*H*_5_/*V*_11_) = 0.9 × 0 = 0, while the nonassigned confidence is *m*(*H*/*V*_11_) = 0.1. In the same way, mass function values about noncore indicator  *V*_12_ over the fuzzy set *H* are *m*(*H*_1_/*V*_12_) = 0.9 × 0.25/0.35 × 0.3 = 0.1929, *m*(*H*_2_/*V*_12_) = 0.9 × 0.25/0.35 × 0.2 = 0.1286, *m*(*H*_3_/*V*_12_) = 0.9 × 0.25/0.35 × 0.3 = 0.1929, *m*(*H*_4_/*V*_12_) = 0.9 × 0.25/0.35 × 0.1 = 0.0643, *m*(*H*_5_/*V*_12_) = 0.9 × 0.25/0.35 × 0.1 = 0.0643, and *m*(*H*/*V*_12_) = 0.357. Mass function about other indicators over the fuzzy set *H* can be calculated as well. Finally, mass matrix about the indicator over the fuzzy set *H* is expressed as

(11) [0.18000.19290.20570.0514    0.3600    0.1286    0.1029  0.1543  0.0900  0.19290.05140.2057  0.2700    0.06430.15430.102900.064300    0.1000  0.3570  0.4857  0.4857].

Complex mass matrix about knowledge source *V*_1_ is derived from evidence combination computation by following computational steps (c) and (d). In the same way, mass matrix about *V*_2_, *V*_3_, and *V*_4_ over the fuzzy set can create a new mass matrix as

(12) $$\begin{array}{c} \left[\begin{array}{ccccc} 0.25 & 0.27 & 0.23 & 0.23 & \;0.02\\ 0.27 & 0.26 & 0.20 & 0.13 & 0.07\\ 0.45 & 0.25 & 0.20 & 0.10 & 0\\ 0.23 & 0.37 & 0.23 & 0.10 & \;0.06 \end{array}\right]. \end{array}$$

Mass matrix about *V* is also obtained by the following computational steps (c) and (d): *m* ^*w*^(*H*_1_/*V*) = 0.27,  *m* ^*w*^(*H*_2_/*V*) = 0.30,  *m* ^*w*^(*H*_3_/*V*) = 0.20,  *m* ^*w*^(*H*_4_/*V*) = 0.16,  *m* ^*w*^(*H*_5_/*V*) = 0.04, and *m* ^*w*^(*H*_5_/*V*) = 0.03.

Studies' results show that experts' high support degree on knowledge network sharing capability among cluster industries is 27%, and relatively high support degree, moderate support degree, relatively low support degree, and low support degree are, respectively, 30%, 20%, 16%, and 4%. The nonassigned confidence is 3%. Based on the maximum membership degree principle, knowledge network sharing capability belongs to relatively high degree.

## 6. Conclusion

Due to asymmetric information, less objective data, and cognitive biases of experts, systematic assessment methods have not dealt with uncertain matters. Evidence Theory is an effective mathematical tool to solve inaccurate and uncertain problems while distinguishing uncertain and unknown facts. Under Dempster's evidence combination rules, experts' opinions with different probability distribution can be operatively synthesized, which bring a new insight to carry out specific assessment.

## Figures and Tables

**Table 1:** Assessment indicator system of knowledge sharing capability in knowledge network.

Principle tier	Subprinciple tier	Assessment class				
		High	Relatively high	Moderate	Relatively low	Low
Assessment index system of knowledge sharing capability in knowledge network (*V*)						
Knowledge Source (*V*_1_, *W*_1_)	Vigor of knowledge (*V*_12_, *W*_12_)	2	4	1	3	0
	Knowledge quantity (*V*_12_, *W*_12_)	3	2	3	1	1
	Interest motive of knowledge sharing (*V*_12_, *W*_12_)	4	2	1	3	0
	Knowledge compatibility (*V*_12_, *W*_12_)	1	3	4	2	0
Path of knowledge transfer (*V*_2_, *W*_2_)	Remunerative transfer of new technology (*V*_21_, *W*_21_)	3	2	3	2	0
	Talent flow (*V*_22_, *W*_22_)	1	4	2	2	1
	Technical corporation (*V*_23_, *W*_23_)	4	2	1	2	1
Carrier of knowledge transfer (*V*_3_, *W*_3_)	Competency of staff (*V*_31_, *W*_31_)	5	2	3	0	0
	Knowledge sharing technology (*V*_32_, *W*_32_)	4	3	1	2	0
Knowledge recipient (*V*_4_, *W*_4_)	Knowledge deficiency (*V*_41_, *W*_41_)	3	3	3	1	0
	Knowledge absorptive capacity (*V*_42_, *W*_42_)	3	2	4	0	1
	Willingness of receiving knowledge (*V*_43_, *W*_43_)	1	6	0	2	1
